# Improving the Characteristics of Fruiting Bodies in *Lentinus edodes*: The Impact of Rolipram-Induced cAMP Modulation

**DOI:** 10.3390/metabo14110619

**Published:** 2024-11-12

**Authors:** Hongman Li, Fei Chen, Chong Xu, Yanhua Wang, Chunhai Deng, Qingguo Meng, Weiwei Zhu

**Affiliations:** Microbial Research Institute of Liaoning Province, Chaoyang 122000, China; lihongman1996@163.com (H.L.); chenfei3033@vip.sina.com (F.C.); sss646@163.com (C.X.); wangyha869@sohu.com (Y.W.); diqiucun2002@163.com (C.D.); goodgege@163.com (Q.M.)

**Keywords:** *Lentinula edodes*, metabolomics, exogenous inducers, fruiting body, gill-free

## Abstract

**Background:** Strains XG04 and XGT2 of *Lentinus edodes* (Berk.) Singer demonstrate a high degree of genomic similarity, with XGT2 representing a systematic selection of XG04 and exhibiting enhanced phenotypic traits. **Methods:** An investigation into the differences between these strains was conducted using untargeted metabolomics to identify potential causal factors. Five exogenous inducers were assessed for their relationship with the observed phenotypes, and their impacts on fruiting body characteristics were analyzed. **Results:** Notably, the exogenous inducer rolipram, at a concentration of 0.4%, was found to increase cAMP expression levels in *L. edodes* primordia, which subsequently affected gill development, leading to the formation of gill-free fruiting bodies. Morphological differences between the two strains were evident; XG04 exhibited a spherical morphology with absent gills, rendering it commercially unviable, whereas XGT2 displayed a thicker cap and a more robust stipe, maintaining its characteristic umbrella shape. **Conclusions:** As the concentration of rolipram increased, both cap retraction and gill reduction in XGT2 occurred in a dose-dependent manner. The endogenous cAMP levels in the fruiting bodies were measured before and after rolipram treatment, revealing that the cap retraction and gill reduction in XGT2 progressed in a dose-dependent manner alongside increasing cAMP expression levels. Furthermore, a positive correlation was observed between cAMP levels and rolipram concentration. This study provides a foundation for improving the quality and productivity of mushroom cultivation by manipulating fruiting body characteristics through external stimuli.

## 1. Introduction

*Lentinus edodes* (Berk.) Singer, commonly known as shiitake mushroom, holds significant importance as an edible mushroom with a rich cultivation history and promising market potential [1]. Recent shifts in dietary preferences have spurred the growth of edible mushroom cultivation, particularly for species such as *L. edodes*, Pleurotus ostreatus (Jacq.: Fr.) Kummer, and *Auricularia auricula* (L.ex Hook.) Underwood [2]. Notably, *L. edodes* was the first edible mushroom to surpass production of 10 million tonnes [3]. According to the China Edible Fungi Association, *L. edodes* production is projected to reach 12.95 million tonnes in 2022, constituting 30.68% of total edible mushroom production in China [4].

The *L. edodes* XGT2 cultivar is a variant of the *L. edodes* XG04 strain resulting from natural genetic variation. The original strain, *L. edodes* XG04, is known for its large fruiting body diameter, thin cap, strong disease resistance, and an approximate 85% bioconversion rate, establishing it as a prominent cultivar in the northern regions of China [5]. In contrast, the *L. edodes* XGT2 strain displays a more spherical mushroom shape, thicker cap, and shorter gill length, and possesses superior commercial attributes, yielding over 90% high-quality mushrooms.

In the present research, the homologous *L. edodes* XG04 and XGT2 primordia were analyzed using a non-targeted metabolomics approach to identify metabolic differences, categorize relevant metabolic pathways, and pinpoint external factors associated with the phenotypic traits of *L. edodes*. Treatments were administered at the primordium stage to investigate the interplay between different types of treatment agents, their concentrations, and the resulting characteristics of the fruiting bodies.

## 2. Materials and Methods

### 2.1. Mycelium Culture

*L. edodes* XG04 and XGT2 strains were preserved in the Edible Fungus Breeding Laboratory at the Microbial Research Institute of Liaoning Province. Conserved strains were inoculated onto Potato Dextrose Agar (PDA) medium and incubated at 25 °C for 5–7 days. Subsequently, they were transferred to Potato Dextrose Broth (PDB) liquid medium and cultured on a shaker at 25 °C and 170 rpm for 9 days. The mycelium was harvested and stored in dark culture at 20 ± 2 °C [6].

PDB Liquid Medium Composition: 10 g dehydrated potato powder, 20 g glucose, 0.5 g MgSO_4_·7H_2_O, 2 g peptone, 0.4 g Na_2_HPO_4_·12H_2_O, and 2.6 g KH_2_PO_4_, diluted to 1 L with RO water.

### 2.2. Cultivation Conditions

The growth substrate consisted of a blend of wood chips, bran, lime, and gypsum in a proportion of 88:10:1:1 (*w*/*w*), with a moisture level of 58% ± 2% (*w*/*w*). This substrate was enclosed in polyethylene bags measuring 17 × 45 cm and weighing 1.25 kg each. The bags were sterilized at 125 °C for 2 h, cooled to 25 °C, and then inoculated with liquid spawn. The substrates were cultivated in a controlled environment chamber set at a temperature of 20 ± 2 °C, relative humidity ranging from 90% to 95%, carbon dioxide concentration of 550 ± 50 ppm, and light intensity between 150 and 350 lux.

### 2.3. Metabolite Extraction

Samples are collected once the primordium has reached a specific size range (5 mm ≤ d ≤ 7 mm). The 50 mg primordium samples were accurately weighed, placed in centrifuge tubes, to which 400 µL pre-cooled methanol/water (4:1, *v*/*v*) solution were added. After 30 s of mixing, glass beads were introduced, and the mixture was snap-frozen in liquid nitrogen for 5 min. The samples were subsequently thawed at room temperature and ground using a frozen tissue grinder for 6 min (−10 °C, 50 Hz). After three cycles of grinding, the centrifuge tubes were spun at 12,000 rpm for 10 min at 4 °C. The supernatant was collected, concentrated, and dried to constant weight. The samples were reconstituted in 400 µL 0.02 mg/mL 2-chlorophenylalanine methanol solution, filtered through a 0.22 µm membrane, and prepared for UPLC-MS analysis [7].

### 2.4. LC-MS/MS Analysis

The instrument platform for this LC-MS analysis was Thermo Fisher Scientific (Waltham, MA, USA)’s ultra-high-performance liquid chromatography tandem Fourier-transform mass spectrometry UHPLC-Q Exactive system.

Chromatographic conditions:

The 2 μL samples were separated by HSS T3 column (100 mm × 2.1 mm i.d., 1.8 μm) and then detected by mass spectrometry. Mobile phase A consisted of 95% water + 5% acetonitrile (containing 0.1% formic acid) and mobile phase B consisted of 47.5% acetonitrile + 47.5% isopropyl alcohol + 5% water (containing 0.1% formic acid). Separation gradient: 0–0.1 min; mobile phase B from linear 0% to 5%: 0.1–2 min; mobile phase B from linear 5% to 25%: 2–9 min; mobile phase B linear from 25% to 100%: 9–13 min; mobile phase B linear maintained 100%. During 13.0–13.1 min, the linearity of mobile phase B decreased from 100% to 0%; during 13.1–16 min, the linearity of mobile phase B remained 0%. The flow rate was 0.40 mL/min and the column temperature was 40 °C.

Mass spectrum conditions:

Positive and negative ion scanning mode was used to collect the sample quality spectrum signal, and the quality scanning range was *m*/*z*: 70–1050. Ion spray voltage, positive ion voltage 3500 V, negative ion voltage 2800 V, sheath gas 40 psi, auxiliary heating gas 10 psi, ion source heating temperature 400 °C, 20-40-60 V cyclic collision energy, MS^1^ resolution 70,000, MS^2^ resolution 17,500.

### 2.5. Bioinformatic Analysis

Bioinformatics analyses were executed using the Majorbio Cloud platform (https://cloud.majorbio.com, accessed on 13 October 2022).

### 2.6. Exogenous Substances Treatment

Each group of 10 samples was exposed to varying concentrations of treating agents at 0.1%, 0.2%, 0.3%, and 0.4%, with the control group receiving water. The *L. edodes* primordia were treated with a single spray application upon reaching a growth stage defined as having diameters between 2 mm and 7 mm, with a minimum of 4 primordia per sample.

### 2.7. Determination of cAMP

Samples from primordia were gathered and the levels of cAMP were measured utilizing a microbial cyclic adenosine phosphate (cAMP) ELISA kit (#AD9210) acquired from Wuhan Addy Anti-Biotechnology Co., Ltd. (Wuhan, China).

## 3. Results

### 3.1. Sample Confidence and Metabolite Correlation Analysis

**Figure 1:**
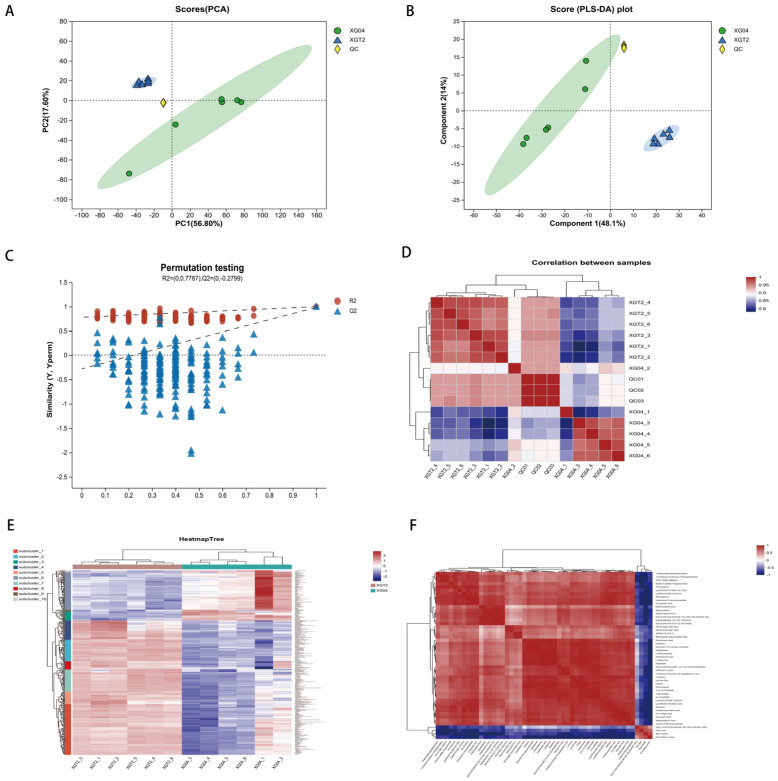
Sample confidence analysis and metabolite correlation analysis. (**A**) PCA score diagram between XG04, XGT2, and QC samples (all sample extracts in equal volume were mixed and used as QC samples). (**B**) PLS-DA score diagram between XG04, XGT2, and QC samples. (**C**) PLS-DA replacement test between XG04, XGT2, and QC samples. (**D**) XG04 sample correlation heat maps between XGT2 and QC samples. (**E**) Heat map of metabolite cluster analysis for XG04 and XGT2. (**F**) Heat map of metabolite correlation for XG04 and XGT2.

Principal Component Analysis (PCA) and Partial Least Squares Discriminant Analysis (PLS-DA) were conducted on the sample data (Figure 1A,B). Samples from the *L. edodes* XGT2 group exhibited greater concentration compared to the XG04 group, indicating higher variability and instability within the XG04 samples. The replacement test (Figure 1C) was successfully executed, validating the reliability of the data. A correlation heat map analysis was performed (Figure 1D), revealing intragroup sample correlations predominantly exceeding 0.9. Inter-group samples differed notably from intragroup samples, highlighting the consistency and reproducibility of the biological replicates [8].

Metabolite cluster analysis was carried out (Figure 1E), demonstrating superior reproducibility in the XGT2 group compared to the XG04 group. Significant metabolic differences were observed between the two strains, with XGT2 displaying a broader range of metabolites and heightened metabolic activity [9]. A pairwise correlation analysis of metabolites with significant differences was conducted (Figure 1F), revealing predominantly positive correlations between XGT2 and XG04 metabolites.

### 3.2. Metabolome and KEGG Analysis

**Figure 2:**
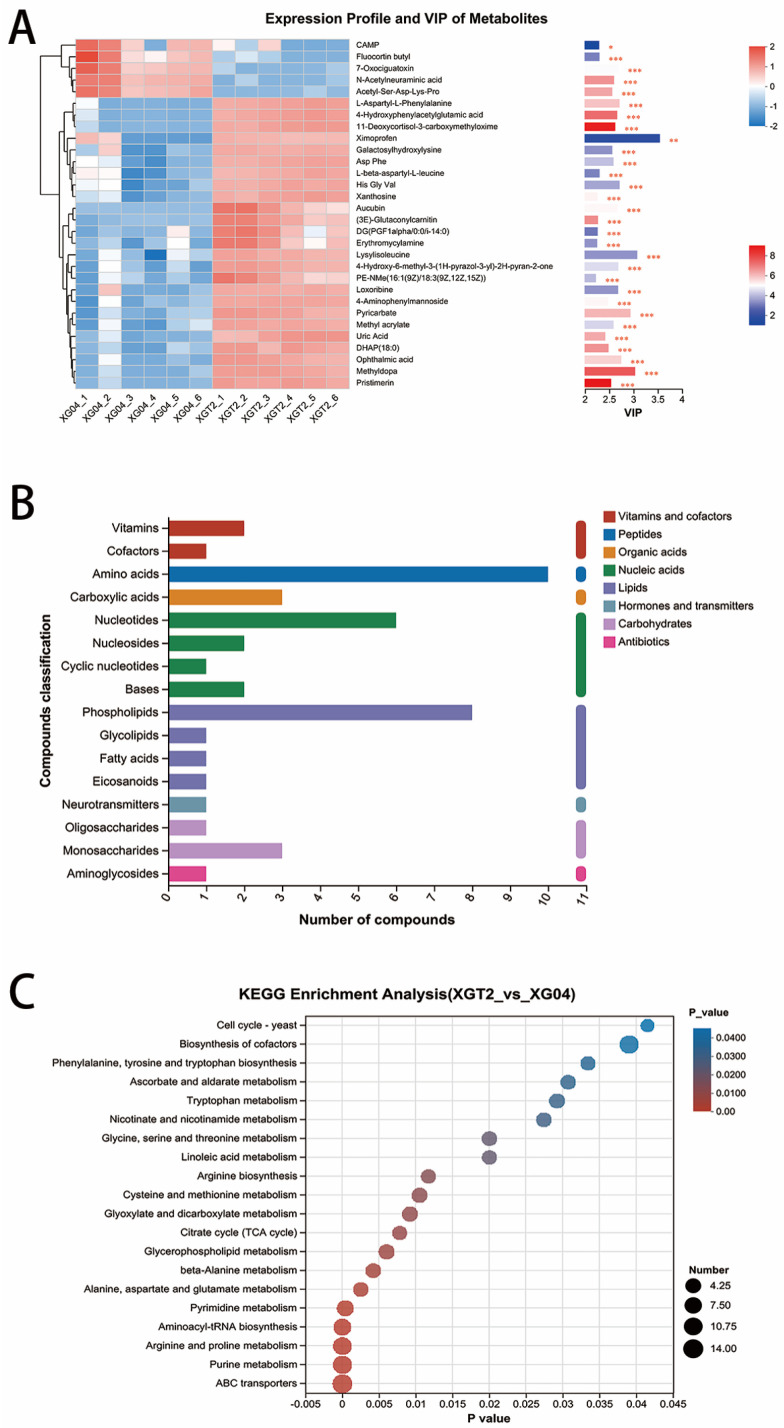
Metabolome and KEGG analysis. (**A**) XG04 and XGT2 metabolite cluster tree and VIP bar diagram. * *p*-value < 0.05; ** *p*-value < 0.01; *** *p*-value < 0.001. (**B**) KEGG compound classification statistical diagram. (**C**) KEGG pathway enrichment analysis bubble diagram.

The metabolite cluster tree and VIP bar chart identified 30 distinct metabolites (Figure 2A). Notably, cAMP, Fluocortin butyl, 7-Oxociguatoxin, N-Acetylneuraminic acid, and Acetyl-Ser-Asp-Lys-Pro exhibited prominent expression in XG04. cAMP, a ubiquitous second messenger, plays pivotal roles in intracellular signaling, hormone activity modulation, and various metabolic pathways [10]. XGT2 showed elevated levels of aucubin, xanthine, losoribine, and uric acid. The conversion of xanthine to uric acid suggests purine metabolism’s significance in *L. edodes* XGT2 [11,12].

KEGG compound statistical classification indicated lipids and nucleic acids as the most abundant metabolites, followed by peptides and carbohydrates (Figure 2B). KEGG pathway enrichment revealed significant associations with ABC transporter proteins, purine metabolism, arginine and proline metabolism, and the tricarboxylic acid (TCA) cycle (Figure 2C), which serves as a metabolic nexus linking sugars, lipids, and amino acids [13].

### 3.3. Phenotypic Modification by Exogenous Substances

The selection of treatment agents was based on the following criteria: metabolite expression differences, pathway enrichment, and VIP metabolite insights. The selected agents for treatment included rolipram, bithionol, xanthosine, aucubin, and allopurinol. The regulation of cAMP concentration is primarily determined by the equilibrium between the synthesis of adenylate cyclase and the hydrolysis of phosphodiesterase (PDEs) [14,15]. Rolipram is a PED4 inhibitor. PDE4 is a specific cAMP hydrolase. Rolipram can elevate the concentration of cAMP by inhibiting the hydrolysis of cAMP. Bithionol is a soluble adenylyl cyclase inhibitor that effectively inhibits soluble adenylyl cyclase by binding to allosteric activation sites, thereby reducing cAMP levels. The expression of xanthosine, aucubin, and uric acid was all significantly elevated in XGT2. Both xanthosine and uric acid are significant products of purine metabolism. ABC transporters represent a pivotal metabolic pathway for xanthosine. Additionally, ABC transporters and purine metabolism are significantly enriched pathways in the KEGG pathway. Aucubin has been demonstrated to promote stem cell regeneration and cell renewal. Xanthosine and aucubin utilize analytical standards to enhance their levels. Uric acid employs a xanthine oxidoreductase inhibitor (allopurinol) to inhibit uric acid synthesis, thereby reducing uric acid levels. With rolipram emerging as the most potent therapeutic agent.

**Figure 3:**
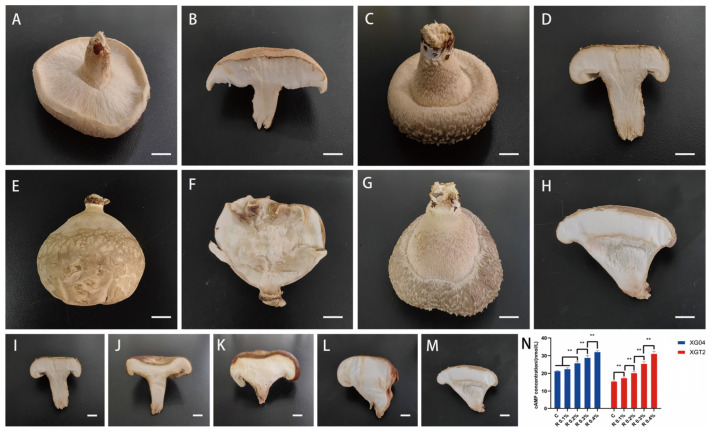
(**A**–**H**) Phenotypic comparison of XG04 and XGT2 under exogenous treatment conditions (treatment: rolipram, concentration: 0.4%). (**I**–**M**) comparison of XGT2 profiles under different rolipram treatment conditions. (**N**) cAMP expression level bar graph. (**A**) XG04 appearance (control). (**B**) XG04 sectional view (control). (**C**) XGT2 appearance (control). (**D**) XGT2 sectional view (control). (**E**) XG04 sectional view (treatment). (**F**) XG04 sectional view (treatment). (**G**) XGT2 appearance (treatment). (**H**) XGT2 section diagram (treatment). (**I**) no treatment. (**J**) 0.1% concentration. (**K**) 0.2% concentration. (**L**) 0.3% concentration. (**M**) 0.4% concentration. Bars: A, B, C, D, E, F, G, H, I, J, K, L, M = 1 cm. (**N**) cAMP levels of XG04 and XGT2 under exogenous treatment conditions (treatment: rolipram, concentration: 0.1%, 0.2%, 0.3%, 0.4%). C: control group. ** *p*-value < 0.01.

Out of the various treatment modalities considered, only rolipram demonstrated efficacy. Under 0.4% rolipram influence, *L. edodes* XG04 and XGT2 fruiting body characteristics were significantly altered (Figure 3A–H). XG04 displayed spherical morphology with absent gills, rendering it commercially nonviable. In contrast, XGT2 exhibited a thicker cap and sturdier stipe, retaining its typical umbrella-shaped form. As rolipram concentrations increased, XGT2 cap retraction and gill reduction progressed in a dose-dependent manner (Figure 3I–M).

Endogenous cAMP levels in fruiting bodies were assessed pre- and post-rolipram treatment (Figure 3N). Elevated cAMP expression was observed in treated groups compared to controls. Between the control group of the two samples, XG04 showed large gills and XGT2 showed small gills. There were individual differences in cAMP level between the two samples, and XG04 was higher than XGT2. This indicates that the size of gill might be correlated with cAMP expression level. XG04 may be more sensitive to cAMP than XGT2. In the XGT2 group, as cAMP expression level increased, XGT2 cap retraction and gill reduction progressed in a dose-dependent manner. Moreover, cAMP levels positively correlated with rolipram concentration.

## 4. Discussion

### 4.1. cAMP Signaling Pathway and Meiosis Process

It has been demonstrated that the intracellular concentration of cyclic adenosine monophosphate (cAMP) in *L. edodes* exhibits fluctuations at different stages of mycelial growth and development, culminating in the formation of fruiting bodies. The condensed mycelia of *L. edodes* exhibited a ninefold increase in adenosine 3′,5′-cyclic monophosphate (cAMP) accumulation in comparison to the non-aggregated mycelia (control) at the onset of fruiting. The immature fruiting bodies also contained cAMP, at a concentration 7.9 times that of the control, while the cAMP content was low in mature fruiting bodies [16]. The accumulation of cAMP in *L. edodes* mycelia was observed to occur steadily up to the stage of condensed mycelial development, which occurred just prior to the onset of fruiting body formation. At the stage of fruiting body maturation, cAMP appears to be actively metabolized, with a notable decrease in intracellular content compared to that observed in condensed mycelia. During the growth and development of the fruiting bodies of *L. edodes*, the changes in cAMP content and metabolic processes were in a balanced, steady state. In this study, the addition of rolipram disrupted this equilibrium, increasing the content of cAMP by inhibiting the hydrolysis of cAMP. This resulted in the remaining cAMP, that could not be metabolized, exerting other physiological effects on the fruiting bodies of *L. edodes*.

cAMP is recognized as a signaling molecule that plays a crucial role in the development of fruiting bodies in basidiomycetes [17,18,19]. The primary pathway responsible for cAMP signaling involves (i) the detection of an extracellular signal via a G-protein coupled receptor; (ii) the subsequent stimulation of the Ga-subunit within a heterotrimeric G-protein; and (iii) the activation of adenylate cyclase, the enzyme responsible for synthesizing cAMP. The binding of cAMP to the regulatory subunit of PKA results in the dissociation of the regulatory subunit from the catalytic subunit (PKAc). The active PKAc then controls protein activities via the phosphorylation of conserved Ser and Thr residues [20]. Unconventional fruiting bodies of *Schizophyllum commune* gills were produced by implementing interventions aimed at increasing intracellular cAMP concentrations. These interventions involved the administration of extracellular cAMP and the use of caffeine to impede phosphodiesterase activity, which degrades cAMP [21,22]. However, a thorough examination of the root causes of this occurrence has not been undertaken, and the connection between this phenomenon and the cAMP signaling pathway has not been investigated through a bioinformatics lens.

Rolipram is an enzyme inhibitor of cAMP hydrolysis, selected from the synthetic and metabolic pathways of cAMP. It is a phosphodiesterase inhibitor that increases intracellular cAMP levels by inhibiting enzyme activity. Oocytes in most species go through a long period of stagnation before meiosis [23,24,25,26,27,28], and cAMP plays an important role in this process. In this study, cAMP inhibits cell meiosis by regulating the synthesis of Moloney Sarcoma Oncogene (MOS) and the activity of MAPK through the cAMP/PKA signaling pathway. The high level of cAMP prevents the accumulation of MOS protein through the cAMP/PKA signal transduction pathway, thus affecting the expression of MOS protein, resulting in the recovery of MAPK signal cascade being hindered [29]. The cAMP/PKA signaling pathway can also directly inhibit MAPK activation [30,31,32]. The smooth progression of meiosis depends on the release of MAPK. Therefore, the inhibition of MOS protein expression and MAPK activation both lead to meiosis arrest. The split gills of *L. edodes* typical are formed by the infolding of the edge of the hymenium. Karyogamy and meiosis occur in an asynchronous manner during this process [33]. High levels of cAMP inhibit the meiosis of the basidial cells, resulting in a cleavage-free gill fruiting body.

### 4.2. Analyzing Metabolic Pathways and cAMP-Associated Processes

**Figure 4:**
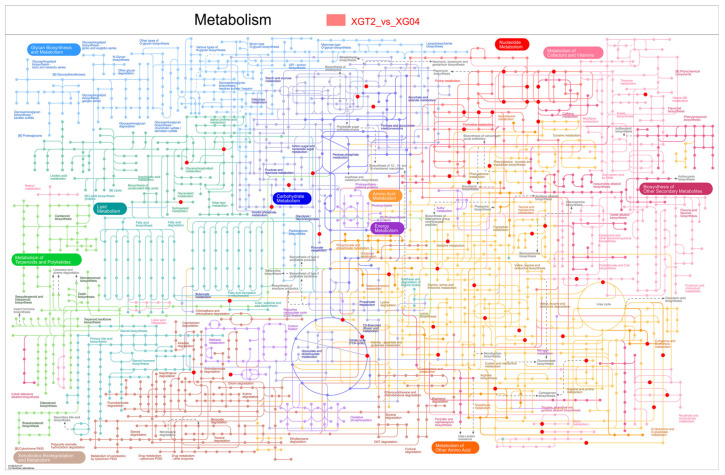
iPath metabolic pathway map.

The metabolic pathways of *L. edodes* XGT2 and XG04 were comprehensively visualized and analyzed (Figure 4). Primary metabolic processes encompass lipids, amino acids, carbohydrates, and nucleotides. Notably, cAMP-associated pathways predominantly influence nucleotide and lipid metabolism. Within nucleotide metabolism, pyrimidine metabolism regulates cAMP synthesis and degradation through reactions like “ATP + CMP ↔ ADP + CDP”, mediated by various kinases such as cmk, panC-cmk, CMK1, CMK2, and UMPK. Meanwhile, glycerophospholipid metabolism plays a pivotal role in lipid metabolism, affecting cAMP synthesis and degradation via CRLS-mediated reactions like “Phosphatidylglycerol + CDP-diacylglycerol ↔ Cardiolipin + CMP.” This surge in cAMP leads to increased intracellular ATP consumption, thereby fueling positive reactions in pyrimidine metabolism.

Research indicates that the uptake of nutrients into the fruiting body occurs in a demand-driven manner rather than being dictated by supply [34]. The process of cell differentiation necessitates substantial reserves of ATP, leading to a competitive dynamic between catabolic and synthetic pathways for energy resources. Extended periods of elevated intracellular cAMP levels can impair the efficacy of second messengers, diminishing their responsiveness to external signals. Additionally, meiosis requires ATP hydrolysis to provide the necessary energy, with critical enzymes involved in this division—such as DNA helicase, DNA polymerase, and RNA polymerase—being ATP-dependent [35]. The consumption of ATP by the cAMP metabolic pathway within the sample adversely affects the meiosis process [36]. The development of fruiting bodies is intricately linked to meiosis, and maintaining ATP homeostasis in fungi is essential for both the timing of fruiting body development and the meiotic process [37]. Any disruption in ATP balance can lead to disturbances in metabolic and signaling pathways, thereby affecting the developmental timing of the fruiting body and resulting in notable phenotypic alterations.

## 5. Conclusions

This research utilized a non-targeted metabolomics methodology to identify metabolic variations and categorize pathways among the closely related strains XG04 and XGT2 of *L. edodes*. Specific exogenous inducers associated with distinct phenotypes were identified for application. The induction of rolipram was observed to enhance cAMP expression in primordia while simultaneously inhibiting gill development. Notably, the application of 0.4% rolipram resulted in the production of gill-free fruiting bodies. These results highlight the potential for manipulating the traits of *L. edodes* fruiting bodies, thereby promoting the efficient and high-quality cultivation of this species.

## Data Availability

The original contributions presented in the study are included in the article, further inquiries can be directed to the corresponding author.
